# Psychosocial distress is a stronger correlate of psychiatric symptom burden than cannabis dependence in young adults: a functional connectome and structural equation modeling study

**DOI:** 10.3389/fnins.2026.1922823

**Published:** 2026-10-28

**Authors:** David Mattie, Jacob Levman

**Affiliations:** 1 Department of Marketing and Enterprise Systems, St. Francis Xavier University, Antigonish, NS, Canada; 2 Department of Computer Science, St. Francis Xavier University, Antigonish, NS, Canada; 3 Research, Innovation and Discovery Center for Clinical Research, Nova Scotia Health Authority, Halifax, NS, Canada

**Keywords:** cannabis, functional connectome, Human Connectome Project, network-based statistic, psychiatric symptom burden, structural equation modeling

## Abstract

**Introduction:**

Cannabis is widely used among young adults and often viewed as a primary driver of adverse psychiatric and neural outcomes, yet the psychosocial and socioeconomic context in which use occurs is rarely modeled jointly with neural and behavioral measures.

**Methods:**

Using the Human Connectome Project young-adult sample (analytic *N* = 1,010–1,016), we employed structural equation modeling to estimate the relative contributions of cannabis, latent psychosocial distress, socioeconomic status, and familial psychiatric risk to three indices of psychiatric symptom burden, while adjusting for polysubstance, alcohol, and nicotine exposure and for family clustering. Cannabis exposure was operationalized in parallel as DSM-IV dependence and as heavy use. Resting-state functional connectomes were examined using graph metrics and network-based statistics, and finite mixture models were used to test for discrete vulnerable subgroups.

**Results:**

Psychosocial distress was the dominant predictor of psychiatric symptom burden across all three outcomes (standardized β = 0.41–0.70), substantially exceeding that of cannabis (total β = 0.05–0.19), which reached significance for only one outcome under the dependence definition. Larger unadjusted group differences (Cohen's *d* = 0.28–0.75) attenuated sharply once distress and demographic factors were modeled. Lower socioeconomic status and greater familial psychiatric risk were each associated with elevated distress, whereas cannabis was not (all indirect β ≤ 0.01, all confidence intervals spanning zero). Neither graph metrics nor the network-based statistic revealed an association between cannabis dependence and the functional connectome, despite approximately 80% power to detect *d*≈ 0.30, and the fitted finite mixture models did not identify a robust and interpretable cannabis-vulnerable subgroup.

**Discussion:**

In a large young-adult sample, psychosocial context rather than cannabis exposure accounted for most of the explained variance in psychiatric symptom burden, and no cannabis-related signature was detectable in the functional connectome. The cross-sectional design cannot establish causal ordering, and because the distress and symptom measures share self-report method variance that the coarse binary exposure measures do not, the relative magnitudes should not be interpreted as a comparison of aetiological importance. These findings nonetheless suggest that risk models and neuroimaging analyses that omit psychosocial context may attribute to cannabis variance shared with distress, disadvantage, and familial risk.

## 1 Introduction

Cannabis is increasingly perceived as a low-risk substance (Camchong et al., 2019) and is commonly used by young adults for self-medication of stress, sleep difficulties, and other discomforts (Wallis et al., 2022). Nearly 37% of Americans report lifetime use (SAMHSA, 2019), and almost half of Canadian young adults (20–24) consumed cannabis within the past year (StatsCan, 2024). Many are chronic users who do not perceive a need to reduce consumption or seek treatment, despite accumulating evidence that heavy exposure is associated with altered brain network efficiency (Xu et al., 2024), disrupted structural connectivity (Maleki et al., 2024), and a range of adverse cognitive and psychiatric outcomes.

However, prior literature reveals substantial heterogeneity in these outcomes (Scott et al., 2017). Some individuals with heavy exposure appear unaffected (Bourke et al., 2019; StatsCan, 2024; Rabiee et al., 2023), whereas others experience impairments in attention, memory, and executive control (Huang et al., 2025; Schantell et al., 2024), and mental health, including elevated risk for depression, anxiety, and psychosis (Colyer-Patel et al., 2024). This variability suggests that cannabis exposure alone is insufficient to explain divergent trajectories and that contextual moderators play an important role in shaping individual outcomes.

Several factors may contribute to this heterogeneity. At the neural level, chronic cannabis use has been linked to altered functional connectivity across large-scale networks, including the default mode, executive control, and salience networks (Ramaekers et al., 2022), as well as white matter abnormalities in tracts connecting prefrontal, limbic, and cerebellar regions (Maleki et al., 2024). Young adulthood (ages 22–35) is a period of ongoing white matter maturation, with long-tract systems such as the cingulum not reaching peak integrity until the early thirties (Lichenstein et al., 2022), making this developmental window particularly relevant to cannabis-related neural effects. Gender differences in psychiatric vulnerability and brain connectivity following cannabis exposure are also well documented (Laurikainen et al., 2019).

Beyond neurobiology, socioeconomic status (SES) is an established determinant of both brain development (Baysarowich et al., 2025; Brito and Noble, 2014) and behavioral health (Rabiee et al., 2023), yet remains underexplored as a moderator in cannabis neuroimaging research. Similarly, family history of psychiatric illness is a recognized risk factor for both substance use and mental health difficulties (Kendler et al., 1997), potentially manifesting as altered connectivity in frontoparietal and cingulate circuits (Baker et al., 2014; Chai et al., 2016; Qin et al., 2022). Psychosocial distress, operationalized via perceived stress, life satisfaction, and sleep quality as indicators of diminished subjective wellbeing (Diener, 1984), and deliberately specified using non-psychiatric indicators to preserve separation from outcome variables (Keyes, 2005), may compound the associations between cannabis exposure and adverse outcomes, or may itself be a stronger predictor of psychiatric burden than cannabis use alone.

Despite the breadth of this literature, no single investigation, to the best of our knowledge, has tested the combined contributions of connectome-level brain efficiency, psychosocial distress, socioeconomic status, and familial psychiatric risk within a unified model of cannabis-related outcomes. Most studies characterize neurobiological differences in isolation from social and contextual factors, and few have assessed whether distinct subpopulations of cannabis users differ meaningfully in their associations with psychiatric outcomes. This isolation is consequential, because analyses that omit the psychosocial context could conceivably attribute variance to cannabis that is more properly attributable to distress, disadvantage, or inherited risk. Resolving the uncertainty between a cannabis-centric account and a psychosocial-context account therefore requires an integrated framework that models these factors together.

In the present study, we employed data from the Human Connectome Project to examine the relative contributions of cannabis exposure, resting-state functional connectome efficiency, psychosocial distress, socioeconomic status, and familial psychiatric risk to cognitive, psychiatric, and behavioral outcomes in young adults. We employed structural equation modeling to estimate these contributions while controlling for polysubstance use and for the family-based clustering inherent to the sample, and we employed finite mixture modeling to assess whether subgroups with distinct cannabis-outcome associations could be identified. To avoid the arbitrariness of a frequency-based threshold, cannabis exposure was operationalized primarily as DSM-IV cannabis dependence, with a heavy-use criterion (≥1, 000 lifetime occasions) retained as a robustness check. Rather than framing the analysis around directional predictions of cannabis-related harm, we treated the cannabis-centric and psychosocial-context accounts as competing explanations that produce divergent expectations. The former implies that cannabis exposure contributes substantially to psychiatric symptom burden and that vulnerable or resilient subgroups are discernible, whereas the latter implies that psychosocial distress, socioeconomic disadvantage, and familial risk account for the majority of explained variance and that any apparent subgroup structure reflects psychosocial heterogeneity rather than differential susceptibility to cannabis. First, we assessed the contribution of cannabis exposure to psychiatric symptom burden relative to, and independent of, psychosocial distress, socioeconomic status, and familial risk; second, whether heavy cannabis exposure is associated with connectome efficiency across seven major networks, considered here as an exploratory aim; and third, whether discrete subgroups consistent with a differential-vulnerability account could be identified.

## 2 Methods

### 2.1 Participants

Data were drawn from the Human Connectome Project (HCP) 1200 Subjects Release, a publicly available dataset of neuroimaging and behavioral assessments of healthy young adults aged 22–35 (Essen et al., 2013). Of the 1,206 participants in the full release (54% female), 1,016 had usable resting-state fMRI (rs-fMRI) data. Behavioral data were obtained from the restricted-access portion of the release, which includes detailed self-report measures of substance use, psychiatric symptomatology, cognitive functioning, and psychosocial variables. Our primary analysis used DSM-IV cannabis dependence criteria (*n* = 94 meeting criteria), where participant meets/met DSM criteria for cannabis dependence at some time over his/her lifetime. As a robustness check, participants were classified as heavy cannabis users if they self-reported lifetime cannabis use of ≥1, 000 occasions on the HCP Semi-Structured Assessment for the Genetics of Alcoholism (SSAGA). This threshold identified 88 heavy users (88/1,016 = 8.6% of the rs-fMRI sample); all remaining participants served as the comparison group.

### 2.2 Analysis pipeline

Psychometric tests that survived Benjamini-Hochberg correction for multiple comparisons were subsequently evaluated to characterize the neural correlates of cannabis dependence. The surviving measures were carried forward into four complementary analytic streams, each interrogating a distinct representation of brain organization. First, whole-brain functional connectomes were derived from the Schaefer-400 parcellation (Schaefer et al., 2018), and cannabis-related differences in inter-regional coupling were assessed across the full set of connections using the network-based statistic (NBS) (Zalesky et al., 2010), which identifies connected subnetworks of altered connectivity while controlling the family-wise error rate. Second, subject-level functional connectivity matrices were derived from the resting-state fMRI time series, providing a full pairwise representation of inter-regional functional coupling, whereby graph-theoretic representations were constructed from these functional connectivity matrices, from which network-level topological features were derived. Third, structural equation modeling was performed that included a latent factor indicated by self-reported measures of distress and was regressed on cannabis dependence alongside relevant phenotypic covariates. Fourth, finite mixture modeling was used to test whether the association between cannabis exposure and psychiatric symptom burden was driven by a discrete vulnerable subgroup rather than varying continuously across individuals.

Our models adjust for socioeconomic status, age, sex, polysubstance exposure, alcohol, nicotine, and familial psychiatric risk. The family structure inherent in the HCP data is permuted as exchangeable units for our NBS analysis; the graph theoretic models use a by-family random intercept, and cluster-robust standard errors by family are used in the structural equation model. The coding, direction and scaling of every covariate are given in Table 1. The analysis pipeline is summarized in Figure 1. All software versions used throughout the analysis are listed in Table 2.

**Table 1:** Construction, direction and scaling of derived variables.

Variable	Construction	Scaling	Direction/coding
Cannabis dependence	SSAGA_Mj_Ab_Dep> 0	Binary	1 = met DSM-IV criteria at any point in lifetime
Heavy cannabis use	SSAGA_Mj_Times_Used≥ band 5	Binary	1 = ≥1,000 lifetime occasions
Perceived stress	NIH Toolbox Perceived Stress, unadjusted	*z*-scored	Higher = more stress
Life dissatisfaction	NIH Toolbox Life Satisfaction, unadjusted	*z*-scored, sign reversed	Higher = less satisfied
Sleep quality	PSQI global score	*z*-scored	Higher = poorer sleep
Socioeconomic status	Mean of *z*-scored income, education and employment status	Mean of three *z* scores	Higher = higher SES
Familial psychiatric risk	Sum across 16 binary parental-history items (8 conditions × 2 parents)	Summed, then *z*-scored	Higher = greater familial loading
Polysubstance exposure	Number of the 5 non-cannabis drug classes (cocaine, hallucinogens, opiates, sedatives, stimulants) for which the participant falls in the highest SSAGA band	Count 0–5, then *z*-scored	Higher = more classes used repeatedly^a^
Alcohol	Drinks per drinking day over the past 12 months (SSAGA ordinal band 0–6)	log(1+*x*), then *z*-scored	Higher = heavier use^b^
Nicotine	Cigarettes per day when smoking regularly (SSAGA midpoint estimate)	log(1+*x*), then *z*-scored	Higher = heavier use
Sex	HCP Gender field	Binary	1 = male, 0 = female

^c^The SSAGA lifetime-use fields are irregularly banded rather than continuous counts, so the highest band corresponds to more than 5 lifetime uses for cocaine, opiates and sedatives, and more than 10 for hallucinogens and stimulants.

^a^The measure is therefore a count of drug classes used repeatedly, not a count of occasions.

^b^The alcohol field is banded rather than a count of drinks: bands 0–4 are single drinks, 5 = 5–6 drinks and 6 = 7 or more, so the top of the scale is compressed.

SSAGA_Mj_Times_Used is banded as 0 = never, 1 = 1–5, 2 = 6–10, 3 = 11–100, 4 = 101–999, 5 = ≥1,000 lifetime occasions.

All *z*-scoring was performed across the analytic sample before model fitting.

We computed familial psychiatric risk and polysubstance exposure as sums and counts, excluding missing items, so a participant with no recorded family history and one with entirely missing family-history data both receive a score of zero; we report the number of affected participants in Table 10. Alcohol and nicotine were recoded such that a missing amount was treated as zero consumption, because these fields are structurally missing for non-users.

**Figure 1:**
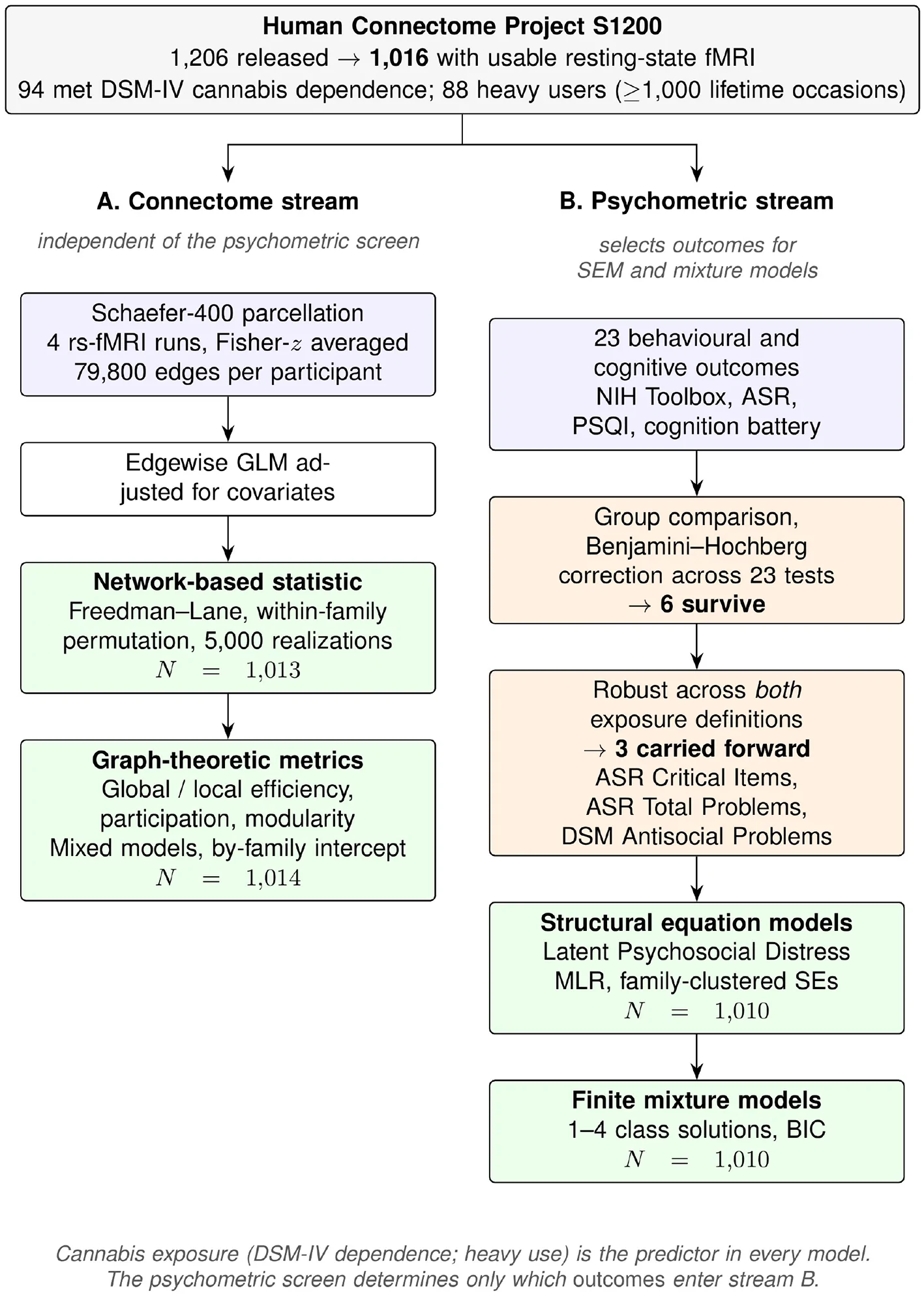
Analysis pipeline. Stream A operates on the resting-state functional connectomes, taking cannabis exposure as the predictor and connectome properties as the outcome; it is not conditional on the psychometric screening step. Stream B screens 23 behavioral and cognitive outcomes for group differences, and the three outcomes that survived correction under both exposure definitions are carried into the structural equation and finite mixture models. The screening step therefore determines which outcomes enter stream B only, and has no bearing on the connectome analyses.

**Table 2:** Software and package versions.

Software	Version	Used for
R	4.6.1	All analyses
targets	1.12.0	Pipeline orchestration and reproducibility
lavaan	0.7.2	Structural equation models
lme4	2.0.6	Linear mixed-effects models
lmerTest	3.2.1	Satterthwaite degrees of freedom
igraph	2.3.3	Connected components and graph metrics
flexmix	2.3.21	Finite mixture models
countreg	0.3.0	Negative-binomial mixture components
effectsize	1.0.3	Cohen's *d* and its confidence intervals
pwr	1.3.0	Smallest detectable effect
openxlsx	4.2.8.1	Reading the HCP phenotype tables

All analyses were run in R (R Core Team, 2026) on x86_64 Linux. The versions listed are those recorded by the pipeline during the run that produced every number reported here. Graph metrics were computed with igraph. Efficiency is the mean of the reciprocal shortest path length over node pairs, local efficiency the mean over nodes of the efficiency of the neighbor subgraph, participation coefficient the standard Guimerà-Amaral formulation on weighted degree, and modularity the Newman modularity of the a priori Yeo seven-network partition. In the primary graph-theoretic analysis and in the proportional-threshold sensitivity analyses (Section 3.4), connection strengths are converted to distances by the reciprocal transform before shortest paths are computed. Structural equation models were estimated under lavaan 0.7.2; every reported fit index, *R*^2^, and path coefficient was reproduced under lavaan 0.6-19 to the precision reported in Table 3.

### 2.3 Behavioral and self-report measures

Self-reported emotional, social, and clinical functioning was assessed through a set of questionnaire-based instruments. Affective and social well-being were measured using the NIH Toolbox Emotio battery (Salsman et al., 2013), comprising satisfaction with life, meaning and purpose, positive affect, friendship, loneliness, perceived hostility, perceived rejection, emotional support, instrumental support, perceived stress, and self-efficacy. Psychopathology was indexed using the Achenbach Adult Self-Report (ASR) (Achenbach and Rescorla, 2003), from which the total problems, internalizing, aggressive behavior, and critical items scales were drawn, together with the DSM-oriented scales for depressive, anxiety, somatic, avoidant personality, attention-deficit/hyperactivity, and antisocial personality problems and the corresponding inattention and hyperactivity subscales. Sleep quality was assessed using the global score of the Pittsburgh Sleep Quality Index (Buysse et al., 1989). A single latent factor, Psychosocial Distress, was defined by three indicators: Perceived Stress (NIH Toolbox Perceived Stress Survey; loading fixed at +1), Life Satisfaction (NIH Toolbox General Life Satisfaction Survey; reverse-keyed, free loading), and Sleep Quality (Pittsburgh Sleep Quality Index global score, higher values denoting poorer sleep; free loading). This specification captures shared variance across a general dysphoria dimension, encompassing subjective stress, diminished wellbeing, and poor sleep rather than any single indicator alone (Yang et al., 2022).

### 2.4 Cognitive tests

Cognitive performance was assessed using the cognition battery of the NIH Toolbox (Denboer et al., 2014), administered as part of the Human Connectome Project protocol. Episodic memory was indexed by the Picture Sequence Memory Test, inhibitory control and selective attention by the Flanker task, and cognitive flexibility by the Dimensional Change Card Sort. Fluid intelligence was measured as the number of correct responses on the 24-item Penn Progressive Matrices (Gur et al., 2010). Impulsive intertemporal choice was additionally characterized using the delay discounting task, summarized as the area under the discounting curve for the small ($200) and large ($40,000) reward conditions.

### 2.5 Sociodemographic and exposure covariates

Several additional measures were retained as covariates and exposure variables. Age and sex were included as demographic covariates. We characterized socioeconomic status by employment status, household income, and years of education. Familial psychiatric and neurological risk was operationalized as a z-scored composite from parental history of schizophrenia, depression, bipolar disorder, anxiety, drug or alcohol problems, Alzheimer disease, Parkinson disease, and Tourette syndrome. Polysubstance exposure was quantified as the number of the five non-cannabis drug classes—cocaine, hallucinogens, opiates, sedatives and stimulants—for which the participant fell in the highest SSAGA use band, counted 0–5 and then *z*-scored. Alcohol and nicotine exposure were retained as separate covariates. The number of alcoholic drinks consumed per drinking day over the past twelve months and cigarettes per day when smoking regularly were each log-transformed and *z*-scored, with missing values treated as zero consumption.

### 2.6 Neuroimaging

Resting-state functional MRI data were acquired as part of the HCP 1200 Subjects Release using a Siemens 3T Connectome Skyra scanner with a multiband echo-planar imaging (EPI) sequence. Each participant underwent four rs-fMRI runs, comprising two sessions with opposing phase-encoding directions (left-right [LR] and right-left [RL]), each session containing two 15-min runs (Run 1 and Run 2). Imaging parameters included a multiband factor of 8, TR = 720 ms, TE = 33.1 ms, flip angle = 52°, voxel size = 2.0 mm isotropic, and 72 axial slices. These parameters were selected to optimize spatial resolution, minimize susceptibility artifacts, and maintain high temporal signal-to-noise ratio (tSNR). Phase encoding alternation (RL and LR) was employed to reduce left-right susceptibility biases. All rs-fMRI data underwent the HCP's minimal preprocessing pipeline (Glasser et al., 2013), which includes gradient unwarping, motion correction, EPI distortion correction using spin-echo field maps, ICA-FIX denoising, and surface-based registration to standard space.

Functional connectomes were constructed using the Schaefer 400-parcel cortical atlas (Schaefer et al., 2018), with parcels assigned to the seven canonical resting-state networks defined by Yeo et al. (2011). For each participant, parcel-wise mean BOLD time series were extracted from each of the four resting-state runs (two sessions × two phase-encoding directions, LR and RL). A Pearson correlation matrix was computed separately for each run, Fisher *r*-to-*z* transformed, averaged across the four runs, and back-transformed to a single correlation matrix per participant. The resulting correlation matrix was treated as a weighted adjacency matrix. We set edges with *r* < 0.30 to zero, and the remaining edges retained their correlation values as weights.

### 2.7 Statistical analysis

#### 2.7.1 Group comparison

Participants were categorized based on DSM-IV cannabis dependence criteria (dependent: *n* = 94; non-dependent: *n* = 920; dependence status was missing for 2 participants). Group differences across 23 behavioral and cognitive outcomes were assessed, and two sets of adjusted *p*-values are reported: *p*_adj_ (*t*) from independent-samples *t*-tests that ignore family structure, and linear mixed models *p*_adj_ (*LMM*) fitted with lme4 (Bates et al., 2015) and lmerTest (Kuznetsova et al., 2017), with a by-family random intercept, which accommodates the twin and sibling structure of the sample. Both were corrected across the 23 outcomes with the Benjamini–Hochberg procedure (Benjamini and Hochberg, 1995). To assess robustness, we repeated these comparisons substituting a heavy-use criterion (≥1, 000 lifetime occasions; *n* = 88) for the dependence diagnosis.

#### 2.7.2 Network-based statistics on functional connectomes of dependent and non-dependent users

To localize the effect of cannabis dependence on whole-brain functional connectivity while controlling the family-wise error rate across the connectome, we employed the network-based statistic (Zalesky et al., 2010). Each of the 79,800 unique edge weights of the Schaefer-400 connectome was Fisher *r*-to-*z* transformed before modeling. For each edge, we then fitted a general linear model in which the transformed weight was regressed on cannabis dependence together with the seven nuisance covariates used throughout; age (in years) and sex (coded 0/1) entered unscaled, and the remaining covariates were *z*-scored. The Fisher transform is conventional for correlation-valued edge weights, but it is not inconsequential at this sample size, and we therefore report the untransformed result alongside the primary analysis.

Nuisance covariates were partialled out using Frisch-Waugh-Lovell residualization: both the exposure regressor and the edge weights were residualized with respect to the nuisance design, and the edgewise *t*-statistic for the cannabis term was computed from the residualized quantities, with residual degrees of freedom taken from the full model. This yielded a matrix of covariate-adjusted *t*-statistics with the same dimensions as the connectome.

The test-statistic matrix was thresholded at a primary level of |*t*|>3.1 (two-tailed), and connected components among the surviving suprathreshold edges were identified using the igraph library (Csardi and Nepusz, 2006), with the size of each component defined as the number of edges it comprised [the “extent” measure of Zalesky et al. (2010)]. A null distribution for the *maximum* component size was then constructed across 5,000 permutations under the Freedman-Lane scheme (Freedman and Lane, 1983), in which the revisualized exposure regressor is permuted while the nuisance structure is held fixed; family-wise-error-corrected *p*-values were obtained by referring each observed component size to this maximum-statistic null.

Because the HCP sample contains twins and siblings, we conducted NBS analysis under two permutation schemes. Under *within-family* permutation, the exposure is shuffled only among members sharing a Family_ID. Under *whole-family* permutation, entire families are exchanged as intact units within strata of equal family size, which preserves the dependence structure within each family while permitting exchange between them, following the restricted-exchangeability framework of Winkler et al. (2014).

In our sample, cannabis dependence varied between families, with only 67 of the 430 families in the analytic sample containing both dependent and non-dependent members. A within-family scheme therefore reshuffles the exposure for only 194 of the 1,014 participants. For this reason, whole-family permutation is the better-matched scheme in this design. The analysis was repeated at |*t*|>2.7 and |*t*|>3.5 to assess the stability of the recovered subnetwork.

#### 2.7.3 Network-level graph-theoretic analysis

To identify whether, and in which functional systems, cannabis dependence was associated with the topological organization of the connectome, we derived a weighted graph from each participant's connectome by proportional thresholding, retaining the strongest 30% of edges and setting the remainder, together with any negative correlations among the retained edges, to zero, so that every participant's graph has the same edge density. Edge weights were converted to distances as the reciprocal of the correlation before any path-based metric was computed, so that stronger connections correspond to shorter paths. Within each of the seven Yeo networks (Yeo et al., 2011), we computed global efficiency and local efficiency of the within-network subgraph, and the mean participation coefficient of the network's parcels, where participation is computed on the whole-brain graph as the fraction of each parcel's connection strength that leaves its own network. Modularity is a property of a partition of the whole connectome rather than of any single network, and was therefore computed once per participant, for the Yeo seven-network partition. We estimated a separate linear mixed-effects model for each of the 21 network-by-metric combinations and for whole-brain modularity using lme4 (Bates et al., 2015) with lmerTest (Kuznetsova et al., 2017), which allowed the association with cannabis dependence to be localized to individual networks and metrics. In each model, the metric served as the outcome and cannabis dependence as the predictor of interest, adjusting for the same seven covariates used throughout, with age in years and sex entered unscaled and the remainder *z*-scored; a by-family random intercept (Family_ID) accommodated the twin and sibling structure in the data, and models were fitted by maximum likelihood. The cannabis-dependence coefficient was extracted from each model, with degrees of freedom and *p*-values obtained via the Satterthwaite approximation (Satterthwaite, 1946). To control for multiple comparisons across the 21 network-by-metric tests, *p*-values were adjusted using the Benjamini-Hochberg false discovery rate procedure (Benjamini and Hochberg, 1995), and effects surviving correction at *p* < 0.05 were interpreted as evidence of a network- and metric-specific association.

As sensitivity analyses, the same models were refitted to metrics computed under proportional thresholding at densities of 0.10 and 0.20, and under an absolute threshold of *r*≥0.30 with untransformed correlation weights, under which edge density varies across participants (Section 3.4).

#### 2.7.4 Head motion

In-scanner head motion was summarized as mean framewise displacement (FD) (Power et al., 2012), computed from the rigid-body realignment parameters distributed with the HCP preprocessed data for each of the four resting-state runs as the sum of the absolute frame-to-frame changes in the three translations and three rotations, with rotations converted to millimeters of displacement on a sphere of radius 50 mm, and averaged over all frames of the four runs that were averaged in the connectome. The influence of motion as a covariate was assessed by (1) comparing mean FD between the dependent and comparison groups, using a rank-sum test and a mixed-effects model of log-transformed mean FD with a by-family random intercept, fitted with and without the seven covariates; and (2) fitting the network-based statistic and the graph-theoretic models with log mean FD (*z*-scored) as an additional covariate.

#### 2.7.5 Structural equation modeling

Structural equation modeling (SEM) was used to address the central question of the study, namely whether cannabis exposure retains a direct association with psychiatric outcomes or whether that association may be more parsimoniously accounted for by co-occurring psychosocial distress. We estimated models in lavaan (Rosseel, 2012) using robust maximum-likelihood estimation (MLR), with cluster-robust standard errors computed using Family_ID as the clustering unit to account for the sample's twin and sibling structure. Missing data were handled by listwise deletion on the modeled variables, yielding an analytic sample of 1,010 of the 1,016 participants (99.4%); given the small proportion of excluded cases, this approach was considered unlikely to bias estimates materially.

Psychosocial Distress was specified as a latent factor with three indicators, namely perceived stress, life satisfaction (reverse-keyed), and PSQI sleep quality, with the loading of perceived stress fixed at unity to set the metric of the factor. In the structural model, each outcome was regressed on cannabis exposure, latent Psychosocial Distress, and the seven covariates included in all analyses (SES, age, sex, polysubstance exposure, alcohol, nicotine, and familial psychiatric risk), while Psychosocial Distress was in turn regressed on cannabis exposure, SES, age, and familial psychiatric risk. The cannabis → Distress path was retained in every model so that the indirect (*a*_1_*b*_1_), direct (*c*_1_), and total (*c*_1_+*a*_1_*b*_1_) effects of cannabis exposure could be estimated as defined parameters, with the total effect reported in Table 3. Standard errors for these defined parameters were obtained using the delta method.

**Table 3:** Model fit and cannabis exposure effects across three outcomes.

Exposure	Outcome	χ^2^ (df)	CFI	TLI	RMSEA	SRMR	*R* ^2^	Indirect β [95% CI]	Total β [95% CI]
Cannabis dep	ASR Critical Items	59.2 (22)	0.955	0.923	0.044	0.024	0.438	0.00 [-0.04, 0.05]	0.13 [0.05, 0.21]
Cannabis dep	Global psychopathology	66.4 (22)	0.951	0.916	0.048	0.024	0.518	0.01 [-0.05, 0.06]	0.05 [-0.01, 0.12]
Cannabis dep	Antisocial traits	51.4 (22)	0.954	0.920	0.039	0.023	0.241	0.00 [-0.03, 0.04]	0.07 [-0.03, 0.16]
Heavy use	ASR Critical Items	60.9 (22)	0.954	0.921	0.045	0.024	0.451	0.01 [-0.04, 0.05]	0.19 [0.11, 0.26]
Heavy use	Global psychopathology	66.5 (22)	0.951	0.915	0.048	0.024	0.519	0.01 [-0.04, 0.06]	0.06 [-0.01, 0.13]
Heavy use	Antisocial traits	51.3 (22)	0.954	0.920	0.040	0.023	0.244	0.01 [-0.02, 0.04]	0.09 [0.01, 0.18]

Each row corresponds to one structural equation model, defined by crossing the cannabis exposure definition (DSM-IV cannabis dependence; heavy lifetime use) with the outcome (ASR Critical Items; global psychopathology, indexed by ASR Total Problems; antisocial traits, indexed by the DSM-oriented Antisocial Personality Problems scale).

All models were estimated with robust maximum likelihood (MLR) and cluster-robust standard errors with families as the clustering unit, using 1,010 of 1,016 participants with complete data on the modeled variables. Robust fit indices are reported (CFI, comparative fit index; TLI, Tucker-Lewis index; RMSEA, root mean square error of approximation; SRMR, standardized root mean square residual), together with the scaled model chi-square and its degrees of freedom and the proportion of outcome variance explained (*R*^2^).

The indirect effect is the product *a*_1_*b*_1_ of the cannabis → distress and distress → outcome paths; the total effect is *c*_1_+*a*_1_*b*_1_.

Missing data were handled by listwise deletion, not by full-information maximum likelihood.

No residual covariances among the distress indicators were specified, so all indicator residuals were constrained to be uncorrelated; with three indicators, the measurement component of the model is just-identified, and such covariances would be at best weakly identified. The same specification was estimated for all three outcomes and both exposure definitions, and no paths were removed after fitting; every path described above was retained in every model irrespective of statistical significance. The complete lavaan syntax is provided in the Supplementary material.

This specification was reached by iterative model development from a theory-implied mediation model, following standard practice for latent-variable models with observed covariates (Kline, 2023): distress was represented as a latent rather than an observed construct, theoretically plausible exogenous covariances were freed, and candidate residual covariances were evaluated and not retained. The *outcomes* to which the model was applied were likewise chosen based on the group comparisons reported in Section 3.1, in the same data. Both features place this analysis in what J6¨reskog (1993) terms the model-generating situation, in which a tentative initial model is modified and retested on the same data until it fits acceptably. Every parameter admits a substantively meaningful interpretation, rather than the strictly confirmatory situation, which he describes as rare in practice. Fit statistics and *p*-values obtained from a model developed on the data it is then tested against are optimistic, and the SEM results should be read as exploratory rather than as a confirmatory test of the cannabis effect. We report in the Supplementary material the same models refitted across the full set of 23 candidate outcomes to allow for direct assessment of the consequences of the outcome selection.

#### 2.7.6 Finite mixture modeling

To test whether a discrete subgroup of individuals was differentially vulnerable to cannabis, rather than treating all cannabis dependent users the same, we fitted finite mixture regression models to each significant outcome using the flexmix package (Grün and Leisch, 2008) in R, regressing the outcome on cannabis dependence, perceived stress, life dissatisfaction, sleep quality, age, SES, and polysubstance exposure within each latent class. Candidate models were estimated under Gaussian, Poisson, and negative-binomial component distributions, and the distributional family was selected by inspecting Poisson overdispersion (the ratio of the Pearson χ^2^ to its degrees of freedom); where overdispersion was present, the negative-binomial model was used. We compared one- to four-class solutions, selected the number of classes using the Bayesian Information Criterion (BIC), and assessed classification quality using normalized entropy and the mean maximum posterior probability. Each solution was estimated from ten independent seeds, each with 50 random starts, and the solution with the lowest BIC was retained. Because mixture likelihoods have many local optima, we report the range of BIC across the ten seeds alongside the BIC value, so solutions that depend on starting values are visible. Convergence, class sizes, normalized entropy, and the mean maximum posterior probability are reported for every outcome and every class solution in Table 4.

**Table 4:** Finite mixture model diagnostics for all three outcomes.

*k*	BIC	BIC range across seeds^a^	Entropy^b^	Mean max. post.	Class sizes	Converged^c^
**ASR Critical Items, negative-binomial components**						
1	4500.1	—	—	1.000	1010	yes
**2–4**	**not estimable in this environment** ^d^					
**ASR Critical Items, Poisson components** ^e^						
1	4604.1	—	—	1.000	1010	yes
2	4553.6	4553.6 to 4579.6	0.340	0.785	259/751	yes
3	4593.1	4593.1 to 4608.5	0.346	0.696	140/825/45	no
4	4640.3	4640.3 to 4654.6	0.374	0.646	45/857/76/32	no
**Global psychopathology, Gaussian components**						
1	6851.5	—	—	1.000	1010	yes
2	6906.4	6906.4 to 6908.1	0.301	0.791	991/19	no
3	6955.7	6955.7 to 6968.1	0.226	0.588	69/57/884	no
4	7013.7	7013.7 to 7021.0	0.285	0.579	43/24/291/652	no
**Antisocial traits, Gaussian components**						
1	5844.7	—	—	1.000	1010	yes
2	4606.6	4606.6 to 4760.2	0.717	0.930	659/351	yes
3	−22766.8^f^	−22766.8 to 4606.6	0.872	0.945	—^f^	yes
4	−24095.0^f^	−24095.0 to −21563.9	0.861	0.931	—^f^	no

^a^Each solution was fitted from ten independent seeds, each with 50 random starts; BIC is from the best of the 10, and the range spans the 10.

A wide range means the solution found depends on its starting values. A one-class model has a unique solution.

^b^Normalized entropy, bounded in [0, 1]; higher values indicate better-separated components. Undefined for *k* = 1.

^c^Whether the EM algorithm met its convergence tolerance within flexmix's default limit of 200 iterations. Solutions marked “no” are reported at the final iteration; none is the preferred solution for its outcome.

^d^The negative-binomial mixtures failed to converge for *k*>1 (non-finite value in the optimizer) under all ten seeds, so the multi-class negative-binomial comparison could not be evaluated.

^e^Reported for completeness only. The Poisson specification is inappropriate for this outcome, whose dispersion (Pearson χ^2^/df) is 1.59; the apparent two-class preference reflects a second component absorbing overdispersion rather than genuine latent structure.

^f^Large negative BIC values arise from a component residual variance collapsing toward zero, the unbounded-likelihood pathology of Gaussian mixtures. These solutions are not interpretable; their exact BIC and class composition depend on the starting values and the numerical platform, so class sizes are not reported.

*N* = 1, 010. Each component regresses the outcome on cannabis dependence, perceived stress, life dissatisfaction, PSQI sleep quality, age, SES, and polysubstance exposure.

The two-class solution for antisocial traits separates participants at or just above the T-score floor of 50 (scores 50–52; the first class listed), where 43.5% of the sample sits, from those above it, and is therefore a censoring artifact rather than a vulnerability subgroup.

## 3 Results

### 3.1 Group comparison

Cannabis dependent users (*n* = 94) differed significantly from the comparison group on 8 of 23 behavioral and cognitive outcomes in the family-clustered mixed models after correction for multiple comparisons, and on 6 of 23 under independent-samples *t*-tests that ignore family structure (Table 5). Effect sizes were significant for ASR Critical Items (*d* = 0.75, *p*_*adj*_ < 0.001), antisocial traits (*d* = 0.60, *p*_*adj*_ = 0.007), attention-deficit/hyperactivity problems (*d* = 0.41, *p*_*adj*_ = 0.022), global psychopathology (*d* = 0.40, *p*_*adj*_ = 0.010), hyperactivity (*d* = 0.36, *p*_*adj*_ = 0.022), and fluid intelligence in non-verbal reasoning (*d* = 0.28, *p*_*adj*_ = 0.014), exhibited in Figure 2.

**Table 5:** Group differences across 23 psychometric outcomes under two cannabis-exposure definitions.

	Cannabis dependence			Heavy use		
Outcome	d	*p*_adj_ (t)	*p*_adj_ (LMM)	*d*	*p*_adj_ (*t*)	*p*_adj_ (LMM)
ASR Critical Items	0.75	< 0.001	< 0.001***	0.94	< 0.001	< 0.001***
Antisocial traits	0.60	0.007	< 0.001***	0.69	0.003	< 0.001***
ADHD problems	0.41	0.022	0.001**	0.36	0.073	0.003**
Global psychopathology (ASR Total)	0.40	0.010	0.002**	0.45	0.006	< 0.001***
ASR Aggressive behavior	0.37	0.103	0.004**	0.47	0.063	< 0.001***
Hyperactivity	0.36	0.022	0.004**	0.37	0.063	0.004**
Fluid intelligence (PMAT)	0.28	0.014	0.034*	-0.02	0.922	0.860
Inattention problems	0.28	0.103	0.034*	0.21	0.175	0.078
Somatic problems	0.21	0.284	0.146	0.29	0.109	0.032*
Anxiety	0.18	0.365	0.195	0.24	0.173	0.069
ASR Internalizing	0.16	0.365	0.206	0.25	0.109	0.062
Perceived stress	0.15	0.365	0.407	0.12	0.379	0.324
Cognitive flexibility	0.14	0.365	0.271	-0.00	0.987	0.824
Self efficacy	0.13	0.365	0.325	0.27	0.073	0.037*
Depressive problems	0.12	0.510	0.325	0.22	0.193	0.080
Avoidant personality problems	0.05	0.796	0.620	0.15	0.313	0.211
Inhibition (Flanker)	0.02	0.860	0.801	-0.11	0.379	0.414
Positive affect	-0.02	0.862	0.801	-0.15	0.295	0.179
Impulsivity (delay discounting $40k)	-0.03	0.817	0.777	-0.23	0.086	0.280
Life has meaning and purpose	-0.04	0.796	0.701	-0.05	0.777	0.564
Life satisfaction	-0.06	0.770	0.701	-0.26	0.085	0.031*
Impulsivity (delay discounting $200)	-0.08	0.627	0.801	0.01	0.954	0.477
Episodic memory	-0.13	0.365	0.192	-0.19	0.173	0.174

*d*, Cohen's *d*, signed so that positive values indicate a higher score in the exposed group.

Two sets of adjusted *p*-values are reported: *p*_adj_ (*t*) from independent-samples *t*-tests that ignore family structure, and *p*_adj_ (LMM) from linear mixed models with a by-family random intercept, which accommodate the twin and sibling structure of the sample. Both were corrected across the 23 outcomes with the Benjamini–Hochberg procedure.

Ordered by Cohen's *d* under the dependence definition. Stars reflect the mixed-model result: ^*^p < 0.05, ^**^p < 0.01, ^***^p < 0.001.

The mixed models are the more appropriate analysis and are also the more sensitive: the median family intraclass correlation across outcomes was 0.16, and accounting for it increased the number of outcomes surviving correction from 6 to 8 under the dependence definition and from 3 to 9 under heavy use. Note that fluid intelligence differs in the opposite direction to the psychopathology measures, the dependent group scoring higher.

**Figure 2:**
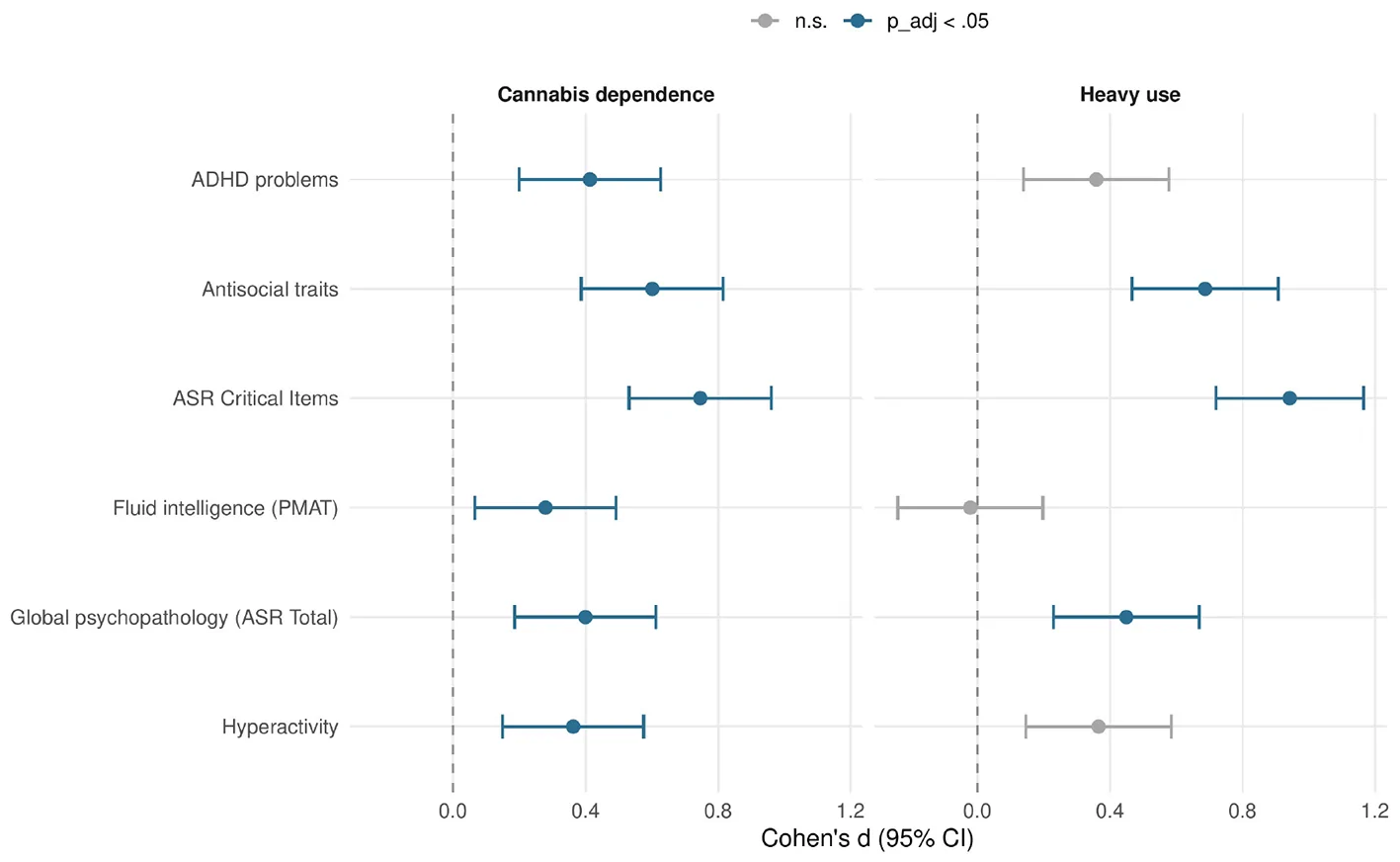
Effect size by psychometric variables with significant group differences after correcting for multiple comparisons.

For the five psychopathology measures, the difference was in the expected direction, with the dependent group scoring higher, except for fluid intelligence, where participants meeting dependence criteria scored *higher* on the Penn Progressive Matrices than the comparison group (18.2 vs. 16.9 correct). This direction was preserved in the structural equation models. We do not interpret this as a benefit of cannabis exposure, and note that it did not replicate under the heavy-use definition (*d* = −0.02, *p*_*adj*_ =0.922); it is reported here for completeness and because the direction is not apparent from effect-size magnitudes alone.

The structural equation models reported below focus on the three outcomes with the largest effect sizes, which were robust across both cannabis-exposure definitions under the *t*-tests: ASR Critical Items, antisocial traits, and global psychopathology. Under the family-clustered mixed models, the set robust across both definitions is larger, comprising those three together with ADHD problems, aggressive behavior, and hyperactivity.

### 3.2 Characterization of cannabis exposure and of the comparison group

Cannabis exposure was operationalized in parallel as DSM-IV dependence and as heavy use. We characterized both groups in detail (Table 6). The two exposure definitions were substantially non-overlapping. Of the 94 participants meeting DSM-IV dependence criteria, 54 (57%) also met the heavy-use criterion, while 40 (43%) did not; conversely, 34 of the 88 heavy users (39%) did not meet dependence criteria. The definitions therefore identify related but distinct populations, consistent with the modest differences in their estimated effects and arguing against treating either as a proxy for the other. All participants meeting dependence criteria reported at least 11 lifetime occasions of use, and 57% reported 1,000 or more.

**Table 6:** Characterization of cannabis exposure and of substance use in the comparison group.

Characteristic	Comparison (n = 920)		Cannabis dependent (n = 94)	
	*n*	%	*n*	%
**Panel A. Lifetime cannabis occasions (SSAGA band)**				
Never used	459	49.9	0	0.0
1–5	212	23.0	0	0.0
6–10	74	8.0	0	0.0
11–100	98	10.7	17	18.1
101–999	43	4.7	23	24.5
≥1,000	34	3.7	54	57.4
**Panel B. Age at first cannabis use (SSAGA band)** ^a^				
≤ 14	34	7.4	30	31.9
15–17	147	31.9	45	47.9
18–20	168	36.4	16	17.0
≥21	112	24.3	3	3.2
**Panel C. Other substances: any lifetime use / repeated use** ^b^				
Cocaine	37	4.0 (1.6)	33	35.1 (23.4)
Hallucinogens	68	7.4 (0.5)	54	57.4 (22.3)
Opiates	38	4.1 (1.1)	48	51.1 (26.6)
Sedatives	34	3.7 (0.8)	31	33.0 (20.2)
Stimulants	55	6.0 (1.5)	35	37.2 (16.0)
**Panel D. Other exposures, mean (SD) or %**				
Polysubstance count (0–5)	0.06 (0.32)		1.09 (1.47)	
of whom count = 0	96.2%		54.3%	
Alcohol, drinks per drinking day (band 0–6)^c^	2.18 (1.54)		3.09 (1.66)	
Regular smokers^d^	21.1%		70.2%	
cigarettes/day if smoker	9.5 (5.9)		10.9 (5.7)	

^a^Percentages in Panel B are of those with a recorded age of first use (461 comparison, 94 dependent). Onset was earlier in the dependent group (Wilcoxon rank-sum *W* = 32, 532, *p* < 0.001).

^b^The first figure is the percentage reporting any lifetime use; the figure in parentheses is the percentage in the highest SSAGA band, corresponding to more than 5 lifetime uses for cocaine, opiates, and sedatives and more than 10 for hallucinogens and stimulants.

^c^SSAGA drinks consumed per drinking day in the past 12 months, banded 0–4 in single drinks, 5 = 5–6 and 6 = 7 or more. Mean among participants with a recorded value (867 comparison, 94 dependent).

^d^Participants with a recorded number of cigarettes per day when smoking regularly (194 comparison, 66 dependent).

Two participants had missing cannabis exposure status and are excluded. The two exposure definitions overlap only partially: of the 94 participants meeting dependence criteria, 54 (57%) also met the heavy-use criterion and 40 (43%) did not; of the 88 heavy users, 34 (39%) did not meet dependence criteria.

Within the comparison group, which comprises every participant not meeting DSM-IV dependence criteria (including the 34 heavy users who did not meet them), half of its members (459 of 920, 49.9%) had never used cannabis, but 175 (19.0%) reported 11 or more lifetime occasions and 34 (3.7%) reported 1,000 or more. Age at first use was substantially earlier in the dependent group, with 32% reporting onset at or before age 14 compared with 7% of the comparison group (Wilcoxon rank-sum *W* = 32, 532, *p* < 0.001).

Use of other substances differed between groups but was uncommon in the comparison group. Any lifetime use of cocaine, hallucinogens, opiates, sedatives, or stimulants was reported by between 3.7% and 7.4% of the comparison group, and repeated use of any given class by between 0.5% and 1.6%; 96.2% of the comparison group had a polysubstance count of zero. The dependent group showed considerably higher use (any use 33.0–57.4%, repeated use 16.0–26.6%), a higher proportion of regular smokers (70.2% vs. 21.1%), and a higher mean number of drinks per drinking day (3.09 vs. 2.18). This pattern indicates the cannabis-dependent group differs from the comparison group in more than cannabis, a point we return to in the Limitations.

### 3.3 Network based statistics

Network-based statistical analysis provided no evidence that a history of cannabis dependence was associated with the functional connectome. Across the 1,014 participants with complete connectivity and covariate data (94 meeting dependence criteria, 920 not), 15 of the 79,800 edges surpassed the primary threshold of |*t*|>3.1 in the covariate-adjusted model. These suprathreshold connections formed only small and fragmented components, the largest comprising three edges spanning four nodes. Across all 15 suprathreshold edges, the split was 11 positive and four negative. The maximum absolute edge statistic across the connectome was 3.55, indicating that although few individual edges reached the screening threshold, their spatial distribution was diffuse and topologically unstructured rather than concentrated within an interconnected subnetwork. No cannabis-related subnetwork was identified, and no nodes were retained for nodal-degree characterization.

Fragmentation remained stable across the primary and sensitivity thresholds. At |*t*|>2.7, the 65 suprathreshold edges formed 15 components; the largest contained 29 edges across 27 nodes. At |*t*|>3.5, only two edges survived, forming two disconnected single-edge components. None of the evaluated thresholds recovered a structure that consolidated into a coherent subnetwork.

No component in the connectome approached significance under any scheme (Table 7). Under the primary whole-family scheme, the observed component of three edges sat far below the null distribution, whose mean maximal component was 181.7 edges and whose 95th percentile was 672.2 edges, giving a family-wise-error-corrected *p* of 0.96. Within-family permutation produced a considerably narrower null (mean 43.3, 95th percentile 174.0). The smallest corrected *p*-value obtained for any component, under any scheme, was 0.92.

**Table 7:** Network-based statistic under two permutation schemes.

Permutation scheme	Observed	Null distribution of maximal component			*p*_FWE_
	(edges)	Mean	95th pct.	Max	
Whole-family (primary)	3	181.7	672.2	23,866	0.964
Within-family	3	43.3	174.0	4,539	0.919

*N* = 1, 014; 5,000 permutations per scheme; primary edge threshold |*t*|>3.1; edge weights Fisher *r*-to-*z* transformed; component size measured as number of edges.

Whole-family permutation exchanges entire families as intact units within strata of equal family size; within-family permutation shuffles only among members of the same family.

### 3.4 Network-level graph-theoretic analysis

Network-level graph-theoretic analysis revealed no association between cannabis dependence and the topological organization of any functional system. Separate mixed-effects models were fitted for global efficiency, local efficiency, and mean participation coefficient within each of the seven Yeo networks, together with a single whole-brain test of modularity, each adjusted for the seven covariates used throughout. Across all 21 network-by-metric tests, the cannabis-dependence coefficient was uniformly small and non-significant (Table 8). Expressed in standard-deviation units of each metric, the largest was 0.13 (visual-network participation), whose unadjusted *p*-value of 0.281 was the smallest of the 21, and no test approached significance after false discovery rate correction (all *p*_*adj*_>0.99); whole-brain modularity was also unrelated to cannabis dependence (*b* = −0.003, *p* = 0.574). The absence of any effect at conventional uncorrected thresholds, before multiple-comparison correction, suggests that this null reflects no detectable association between cannabis dependence and network-level functional topology in this sample.

**Table 8:** Cannabis dependence is unrelated to network-level functional topology.

Network	Global efficiency *b* (*p*)	Local efficiency *b* (*p*)	Mean participation *b* (*p*)
Visual	-0.009 (0.548)	-0.011 (0.506)	0.004 (0.281)
Somatomotor	-0.011 (0.498)	-0.007 (0.646)	-0.004 (0.514)
Dorsal attention	-0.006 (0.552)	-0.007 (0.505)	0.000 (0.909)
Salience/ventral attention	-0.005 (0.608)	-0.004 (0.691)	0.002 (0.687)
Limbic	-0.008 (0.528)	0.001 (0.971)	-0.004 (0.753)
Frontoparietal control	-0.002 (0.802)	-0.003 (0.702)	-0.003 (0.567)
Default mode	0.001 (0.932)	0.000 (0.998)	0.000 (0.946)

Unstandardized cannabis-dependence coefficients (*b*), in the units of each metric, with uncorrected *p*-values from separate mixed-effects models, each adjusting for the seven covariates used throughout (SES, age, sex, polysubstance exposure, alcohol, nicotine, and familial psychiatric risk), with a by-family random intercept. Metrics were computed after proportional thresholding at a density of 0.30, with edge distance defined as the reciprocal of correlation. No effect is significant before or after FDR correction across the 21 tests.

*N* = 1, 014 for every cell except limbic global and local efficiency, which are undefined when fewer than two edges of the limbic subnetwork survive thresholding; this occurs for 67 of the 1,014 participants, so those two cells are based on *N* = 947. Participation is computed on the whole-brain graph and is defined for all participants.

Whole-brain modularity was likewise unrelated to cannabis dependence (*b* = -0.003, *p* = 0.574). In standard-deviation units of each metric, the largest coefficient in the table, for visual-network participation, is 0.13.

The null was not specific to the graph construction (Table 9). Under the absolute threshold, and under proportional thresholding at densities of 0.10 and 0.20, no test survived correction. Of the 100 tests across the four schemes, only one reached nominal significance: a lower limbic local efficiency in the dependent group at density 0.20 (β = −0.41, *p* = 0.005, *p*_*adj*_ = 0.122), which did not replicate at the neighboring densities and falls in the network whose metrics are least stable under thresholding. The coefficients were larger under the sparser proportional schemes (maximum |β| of 0.38 and 0.41 at densities of 0.10 and 0.20) than under the primary scheme. However, none were significant after correction, so that the choice of density influenced the magnitude of the estimates but did not change the conclusion. The whole-brain metrics under every scheme are reported in Supplementary material S3.

**Table 9:** Cannabis-dependence effects on network topology under four thresholding schemes.

Scheme	Density	Tests	Max |β|	Smallest *p*	Surviving BH
Absolute (*r*≥0.30)^a^	0.199 (0.025–0.836)	25	0.218	0.086	0
Proportional, density 0.10	0.100 (fixed)	25	0.381	0.087	0
Proportional, density 0.20	0.200 (fixed)	25	0.412	0.005^b^	0
Proportional, density 0.30^c^	0.300 (fixed)	25	0.134	0.281	0

^a^Absolute threshold with untransformed correlation weights. Density is reported as mean (range) across participants; under the proportional schemes it is fixed by construction.

^b^Limbic local efficiency. It does not survive correction (*p*_*adj*_ = 0.122), does not replicate at densities 0.10 or 0.30, and falls in the least stable of the seven networks. It is the only one of the 100 tests to reach nominal significance. With head motion added as a covariate it is *p* = 0.003, *p*_*adj*_ = 0.070.

^c^The primary analysis, reported in full in Table 8.

*N* = 1, 014, except for limbic global and local efficiency (*N* = 679, 425, 786 and 947 under the four schemes in the order listed), which are undefined when too few limbic edges survive thresholding. Each scheme contributes 21 per-network tests (global efficiency, local efficiency and mean participation coefficient across the seven Yeo networks) plus four whole-brain tests (global efficiency, local efficiency, mean participation coefficient, modularity), corrected together.

Under the three proportional schemes, distance is the reciprocal of correlation. All models include a by-family random intercept, are fitted by maximum likelihood, and adjust for the seven covariates used throughout (SES, age, sex, polysubstance exposure, alcohol, nicotine and familial psychiatric risk). Outcomes are *z*-scored, so coefficients are between-group differences in standard deviation units.

### 3.5 Head motion

Head motion did not differ with cannabis dependence. All 1,014 participants with known exposure status had realignment parameters for all four runs. Mean FD was 0.169 mm (SD 0.062) in the dependent group and 0.164 mm (SD 0.056) in the comparison group (rank-sum *p* = 0.448), and the difference in log mean FD was negligible in a mixed-effects model with a by-family random intercept, whether unadjusted (β = 0.006 SD, *p* = 0.955) or adjusted for the seven covariates (β = −0.17 SD, *p* = 0.142). In the network-based statistic, 14 edges exceeded the primary threshold, the largest component comprised three edges over four nodes, and the maximum absolute edge statistic was 3.61. In the primary graph-theoretic analysis, the largest coefficient was 0.14 standard deviations (visual-network participation, *p* = 0.263) and no test survived correction; across the four thresholding schemes, the limbic local efficiency result at density 0.20 remained the only nominally significant test (*p* = 0.003, *p*_*adj*_ = 0.070).

### 3.6 Structural equation modeling

We estimated a parallel set of structural equation models in which a latent psychosocial distress factor and cannabis exposure were specified as correlated predictors of three externalizing-relevant outcomes, the Achenbach Adult Self-Report Critical Items scale, the ASR Total Problems T-score, and the DSM-oriented Antisocial Personality Problems T-score, with the distress factor permitted to mediate the association between exposure and each outcome. Cannabis exposure was operationalized in two ways across otherwise identical models, as DSM-IV cannabis dependence and, in parallel, as heavy lifetime use, to assess whether findings were sensitive to the exposure definition. We fit all models with a robust maximum likelihood estimator (MLR) and cluster-robust standard errors, clustering by family to account for the non-independence introduced by related and twin participants in the sample. Listwise deletion on the modeled variables yielded an analytic sample of 1,010 of 1,016 participants for each model; the six excluded cases are itemized in the participant-flow table (Table 10). Table 3 exhibits model fit and total cannabis exposure effects across three outcomes. Figures 3a–c show structural equation model plots for each of the three outcomes of interest.

**Table 10:** Participant flow and analytic sample by analysis.

Stage/ analysis	*N*	Excluded	Reason
HCP S1200 full release	1,206	—	—
Usable resting-state fMRI	1,016	190	No usable rs-fMRI
Group comparisons (Table 5)	1,014	2	Cannabis exposure status missing
Graph-theoretic models (Table 8)	1,014	2	Cannabis exposure status missing
Network-based statistic	1,014	2	Cannabis exposure status missing
Head-motion analyses	1,014	2	Cannabis exposure status missing
Structural equation models	1,010	6	Listwise deletion (see note)
Finite mixture models	1,010	6	Listwise deletion (see note)

Missing values across the modeled variables were few and partially overlapping: ASR Critical Items, ASR Total Problems, and DSM Antisocial Problems were each missing for 3 participants, socioeconomic status for 2, cannabis exposure status for 2, and perceived stress and life satisfaction for 1 each. Listwise deletion across the full modeled set therefore removed 6 participants in total, leaving 1,010 of 1,016 for every structural equation and finite mixture model. The 1,010 participants belong to 428 distinct families (the full 1,016 to 430). In the group comparisons, the *N* for an individual outcome ranges from 1,010 to 1,014 because of missing outcome values.

**Figure 3:**
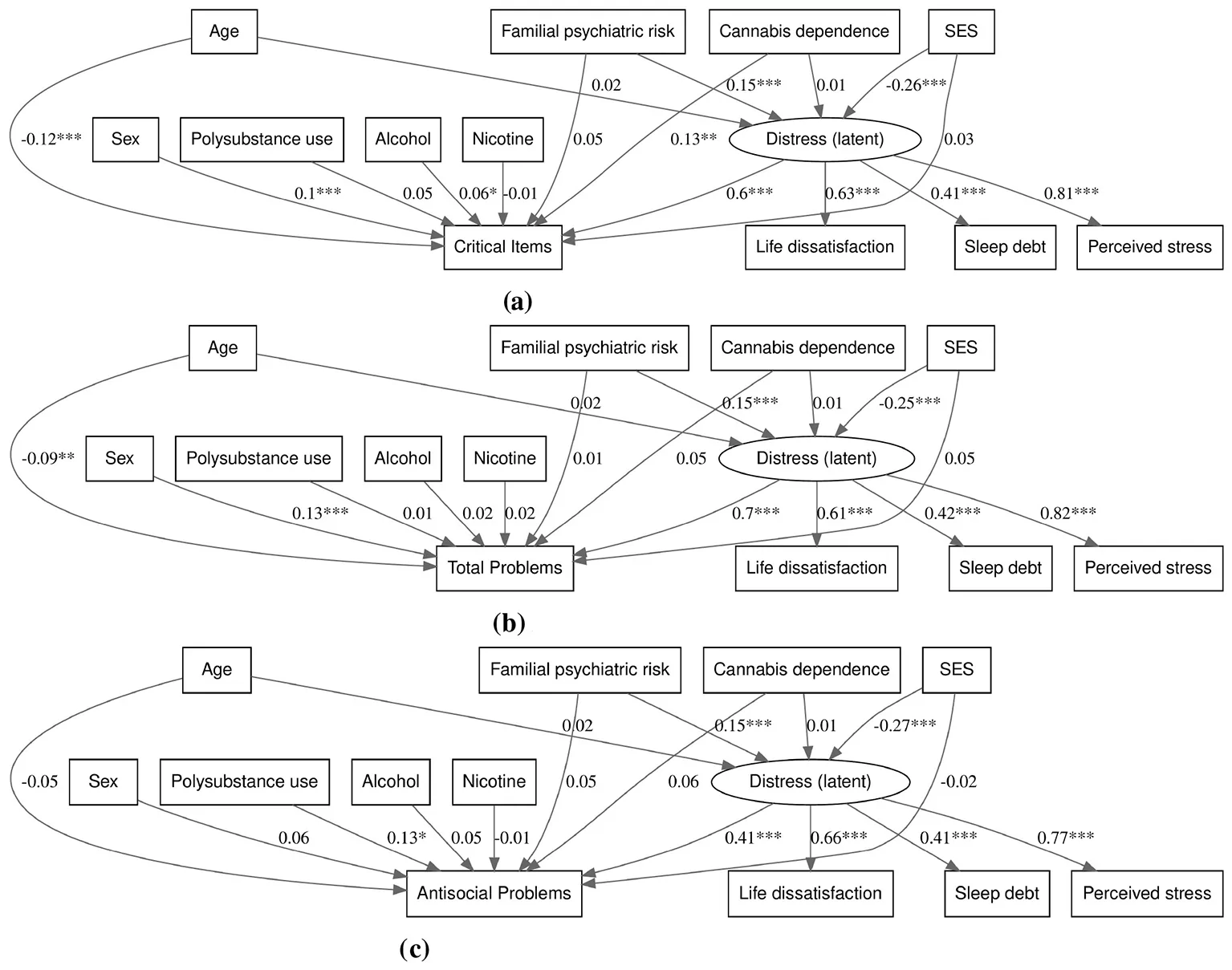
Structural equation models relating cannabis dependence to three psychiatric outcomes. Each outcome is regressed on cannabis dependence, a latent Psychosocial Distress factor, and covariates; latent Distress is defined by perceived stress, PSQI sleep quality, and reverse-keyed life satisfaction. Single-headed arrows are directed regression and measurement paths; values are completely standardized coefficients (β). Models were estimated with robust maximum-likelihood estimation (MLR) and cluster-robust standard errors with Family_ID as the clustering unit, using listwise deletion on the modeled variables. **(a)** ASR critical items. **(b)** ASR total problems. **(c)** DSM antisocial personality problems.

Model fit was acceptable and largely uniform across exposures and outcomes. Robust comparative fit indices ranged from 0.951 to 0.955, robust Tucker-Lewis indices from 0.915 to 0.923, robust RMSEA from 0.039 to 0.048, and SRMR from 0.023 to 0.024. The scaled χ^2^ statistic was significant in every model (χ^2^(22) = 51.3 to 66.5, all *p* < 0.001), which is unsurprising given the sensitivity of the chi-square to sample size at N = 1010 and which we therefore did not treat as evidence of meaningful misfit in light of the close-fit RMSEA and the low SRMR. The latent distress factor was well defined by its three indicators: perceived stress, reverse-keyed life satisfaction, and a PSQI sleep-quality index, with factor loadings ranging from |λ| = 0.41 to 0.82 (all *p* < 0.001), indicating adequate measurement model coherence.

Across all models, psychosocial distress emerged as the dominant correlate of problem burden (Figure 4). The intervals in that figure come from a nonparametric bootstrap resampling of whole families with replacement, 1,000 replicates, reported as percentile intervals; all replicates converged. Delta-method intervals derived from the cluster-robust covariance matrix are near-identical to these, agreeing on all 27 coefficients as to whether the interval excludes zero and differing in mean width by 1%. The standardized path from the distress factor to the outcome was substantial in every case, with values of 0.60 and 0.61 for Critical Items, 0.70 and 0.70 for Total Problems, and 0.41 and 0.42 for Antisocial Problems under the dependence and heavy-use models, respectively (all *p* < 0.001). Removing the distress factor from the outcome equation reduced *R*^2^ by 16.5 to 45.5% across outcomes, whereas removing the cannabis exposure reduced *R*^2^ by less than 2% in every model (0.1% to 1.7%). This pattern indicates that, once distress and the covariate set were accounted for, cannabis exposure contributed little unique predictive information for any of the three outcomes.

**Figure 4:**
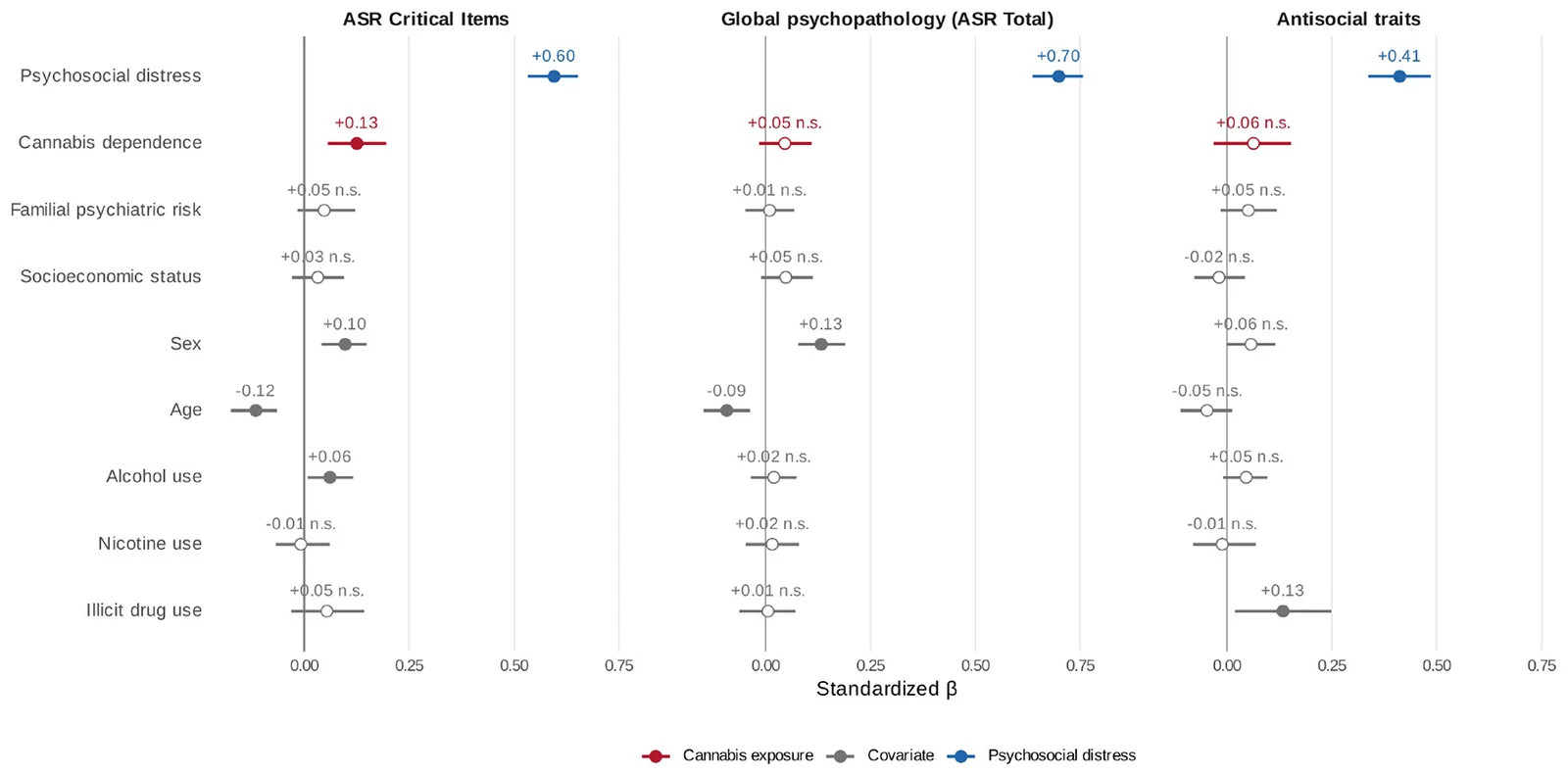
Standardized predictors of three psychiatric outcomes from the structural equation models. Each panel plots the completely standardized regression coefficient (β) for every predictor of one outcome: ASR Critical Items, global psychopathology (ASR Total Problems), and antisocial traits. Points are completely standardized coefficient estimates and horizontal lines are 95% percentile confidence intervals from a nonparametric bootstrap in which whole families, rather than individual participants, were resampled with replacement (1,000 resamples, all of which converged), so that the twin and sibling structure of the sample is preserved in every replicate. The vertical line marks β = 0. Psychosocial distress (the latent factor) and cannabis dependence are highlighted as the two focal predictors, with all remaining variables shown as covariates. Coefficients whose confidence interval includes zero are labeled “n.s.” Across all three outcomes, psychosocial distress is the dominant predictor (β = 0.60, 0.70, and 0.41), whereas cannabis dependence shows a small association (β = 0.13, 0.05, and 0.06 for the direct effect), illustrating that variation in these outcomes is carried substantially more by psychosocial context than by cannabis exposure. The family bootstrap intervals are almost indistinguishable from the delta-method intervals from the cluster-robust covariance matrix (mean width ratio 0.99), and the two agree on whether each of the 27 coefficients excludes zero.

Of particular interest, the mediated pathway was empty. The standardized association between cannabis dependence and the latent distress factor was negligible and non-significant in every model (*a*_1_ = 0.006–0.009 across the three outcomes, all *p*>0.82; for the Critical Items model, *a*_1_ = 0.008, 95% CI [−0.072, 0.088], *p* = 0.84), and the corresponding associations under the heavy-use definition were likewise negligible (*a*_1_ = 0.011–0.015, all *p*>0.68). Because the indirect effect is the product of this near-zero a-path and the substantial b-path, the standardized indirect effects were correspondingly trivial and non-significant in all six models (|β| ≤ 0.007, all *p*>0.70, all confidence intervals spanning zero). We therefore find no evidence that psychosocial distress functions as a pathway linking cannabis exposure to externalizing problems in this sample; rather, distress and cannabis exposure appear to operate as independent correlates of problem burden, with distress the more substantial of the two.

The modest associations that cannabis exposure did exhibit with the outcomes were direct rather than distress-mediated, and were confined to a subset of outcomes. Under the dependence definition, only the Critical Items scale showed a reliable direct association (β = 0.125, *p* = 0.001), while the direct associations with Total Problems (β = 0.046, *p* = 0.14) and Antisocial Problems (β = 0.063, *p* = 0.17) did not reach significance. Under the heavy-use definition, the direct association with Critical Items was somewhat more pronounced (β = 0.179, *p* < 0.001), the association with Antisocial Problems reached significance (β = 0.087, *p* = 0.045), and the association with Total Problems remained non-significant (β = 0.053, *p* = 0.089). The two exposure definitions therefore produced convergent results, with heavy use yielding the broader and slightly stronger direct associations. However, we are reluctant to over-interpret this difference given the overlapping and modestly sized exposed subgroups (94 participants meeting dependence criteria and 88 classified as heavy users). Among the covariates, familial psychiatric risk was consistently and positively associated with the latent distress factor (β≈0.16, *p* < 0.001) across models, and age and sex showed the expected small associations with problem burden (Solmi et al., 2022; McGrath et al., 2023).

These findings should be interpreted in light of the cross-sectional design, which precludes any temporal ordering of cannabis exposure, distress, and problem burden, such that the direct and indirect terms estimated here are decompositions of conditional covariance rather than evidence of a causal sequence; the absence of a mediated pathway is consequently best read as an absence of shared variance between cannabis exposure and the distress factor rather than as a refutation of any particular neurodevelopmental model. The relatively small proportion of participants meeting dependence or heavy-use criteria further constrains statistical power to detect small direct associations of the exposure, even as the large overall sample affords precise estimation of the dominant distress paths.

### 3.7 Sensitivity to outcome selection

The three outcomes carried into the structural equation models were chosen based on the group comparisons reported above, using the same data. To quantify the cost of that selection, we refit the same model specification to all 23 candidate outcomes under both exposure definitions. Two outcomes, perceived stress and life satisfaction, are indicators of the latent distress factor and were necessarily excluded as circular, leaving 21 models per exposure definition.

The selection did inflate the cannabis estimates, as expected. Across all 21 outcomes, the median absolute standardized total effect of cannabis was 0.034 under the dependence definition and 0.048 under heavy use, whereas the corresponding medians among the three published outcomes were 0.066 and 0.093, approximately twice as large. Any reading of the published cannabis coefficients as typical of cannabis-outcome associations in this sample would therefore be mistaken, and we have avoided that reading.

The substantive conclusion nevertheless survived the broader analysis. The cannabis total effect reached significance for only 2 of 21 outcomes under each exposure definition, whereas the distress path reached significance for 17 of 21, and the absolute distress coefficient exceeded the absolute cannabis coefficient in 20 of 21 outcomes. The median absolute distress path was 0.460 across all outcomes, compared with 0.595 among the three published ones, so the distress estimates were inflated by selection considerably less than the cannabis estimates were. The single outcome for which cannabis was the stronger predictor was fluid intelligence, where the distress path was itself negligible and non-significant (β = −0.07, *p* = 0.10) and where, as noted above, the cannabis association ran opposite to a deficit account and did not replicate across exposure definitions. Full results for all 21 outcomes are provided in the Supplementary material.

### 3.8 Finite mixture models

Full diagnostics for all three outcomes and all class solutions are reported in Table 4.

For ASR Critical Items, the outcome was overdispersed relative to the Poisson assumption (dispersion = 1.59). A Poisson mixture favored a two-class solution (BIC_1_ = 4604.1, BIC_2_ = 4553.6, BIC_3_ = 4593.1), but that preference appears to be an artifact of fitting an equidispersed distribution to overdispersed counts such that a second component is recruited to absorb variance the Poisson cannot accommodate. Under the specified negative-binomial component distribution, however, only the one-class model could be estimated, since the negative-binomial mixtures did not converge for *k*>1 under any of the ten seeds; the one-class model is therefore retained, and the multi-class negative-binomial comparison could not be evaluated.

For global psychopathology, a single-class solution was clear, with BIC increasing monotonically across solutions (6851.5, 6906.4, 6955.7, 7013.7). The best two-class alternative did not describe a subgroup of any substantive size: its second component held 19 participants (1.9%), and the EM algorithm had not converged when it reached its 200-iteration limit.

For antisocial traits, BIC favors a two-class solution (5844.7 vs. 4606.6). We believe that solution is an artifact of censoring rather than evidence of a vulnerable subgroup. The DSM-oriented Antisocial Personality Problems T-score has a floor at 50 and 43.5% of participants are at the floor. The two-class solution is also sensitive to its starting values: across the ten seeds its BIC ranged from 4606.6 to 4760.2, and the value reported is the best of the 10. The three- and four-class solutions returned large negative BIC values arising from a component variance collapsing toward zero and were not interpretable.

Taken together, the fitted mixture models did not identify a robust and interpretable cannabis-vulnerable subgroup for any of the three outcomes. These results do not, however, amount to a clean statistical demonstration of a single-class population structure, since the multi-class negative-binomial models for Critical Items could not be estimated and the two-class solution favored by BIC for antisocial traits was set aside on interpretive rather than statistical grounds. We therefore take them as indicating that the fitted models did not identify such a subgroup, not that none exists; mixture models recover latent classes only when components are adequately separated and sufficiently populated, and a subgroup comprising a modest fraction of 94 dependent participants would be difficult to detect under any of these specifications.

## 4 Discussion

The absence of any cannabis-related subnetwork effects appears to be in contrast with existing work reporting altered functional connectivity in cannabis users across multiple networks (Ramaekers et al., 2022). However, the discrepancy is largely reconciled by a difference in what each design measures. The Ramaekers study administered cannabis or Δ^9^-tetrahydrocannabinol (THC) shortly before or during scanning. It contrasted the drug condition against placebo, so that the connectivity differences they recovered index the acute pharmacodynamic action of exogenous cannabinoids on a brain that is intoxicated at the moment of measurement. The present analysis addressed a different question, namely whether a history of cannabis dependence leaves an enduring signature on the resting-state connectome of individuals who were not acutely intoxicated when scanned, the Human Connectome Project protocol having screened for same-day substance use on testing days. Therefore, the divergent findings may indicate that functional connectivity could be reorganized while cannabinoids are pharmacologically active, yet that reorganization does not, in the present data, consolidate into a stable trait-level architecture recoverable from the drug-free resting state at the level of diagnostic status.

The central finding of this study is that psychosocial distress, defined as a latent factor combining perceived stress, diminished life satisfaction, and poor sleep quality, was the strongest and most consistent predictor of psychiatric symptom burden across all three structural equation models, with standardized path coefficients (β = 0.41–0.70) substantially exceeding those of cannabis exposure (total β = 0.05–0.19 across outcomes and exposure definitions). The models explained 24–52% of outcome variance. Lower SES and greater familial psychiatric risk were both associated with elevated psychosocial distress, suggesting that socioeconomic disadvantage and inherited vulnerability converge on a common distress pathway that may represent a closer correlate of psychiatric burden than cannabis exposure itself.

Cannabis exposure was, by contrast, associated with the outcomes only weakly and inconsistently. Under the dependence definition a reliable association was observed for just one of the three outcomes (ASR Critical Items, total β = 0.13), with the confidence intervals for global psychopathology and antisocial traits both spanning zero; under the heavy-use definition, associations reached significance for Critical Items and antisocial traits but not for global psychopathology. The bivariate group differences were considerably larger before adjustment (Cohen's *d* = 0.28–0.75 across the six outcomes that survived correction), which is consistent with much of the apparent cannabis association reflecting co-occurring psychosocial and demographic factors rather than an effect of the substance itself, though the cross-sectional design does not permit that attribution to be tested directly. The outcomes most strongly differentiating cannabis groups spanned a transdiagnostic clinical-severity index (ASR Critical Items), global psychopathology (ASR Total Problems), and a specific externalizing dimension (antisocial traits), with smaller and largely non-robust differences on internalizing measures. The one cognitive measure to survive correction, fluid intelligence, differed in the opposite direction to a deficit account and did not replicate across exposure definitions, and we therefore place no interpretive weight on it. To the extent that an externalizing signal is present, it is carried by the antisocial-traits outcome and is consistent with prior work linking cannabis use to disinhibition and rule-breaking rather than to broad-spectrum psychopathology (Scott et al., 2017). Notably, cannabis exposure was not significantly associated with the latent psychosocial distress factor itself, and the resulting indirect effects were correspondingly negligible in every model (all |β| ≤ 0.01, all confidence intervals spanning zero), indicating that cannabis was not measurably related to underlying distress in this sample. This dissociation, set against widespread self-reported use of cannabis for stress relief (StatsCan, 2024; Wallis et al., 2022), suggests that whatever acute effects motivate such use do not translate into durable differences in subjective distress at the level of dependence or heavy use.

The neuroimaging analyses returned no evidence that cannabis dependence is associated with the functional connectome. Neither the network-level graph-theoretic analysis, which found cannabis-dependence coefficients to be small and non-significant across all twenty-one network-by-metric tests and for whole-brain modularity, nor the whole-brain network-based statistic, which recovered no connected component surviving family-wise correction, identified a reliable cannabis-related signature. That two analytic approaches operating at different levels of detail, one summarizing the topology of predefined functional systems and the other searching the full edge set for spatially contiguous effects, converged on the same null is informative, and the null held under four graph constructions and after adjustment for in-scanner head motion, which did not differ between the groups. This convergence suggests that the absence is not readily explained as an artifact of the analytic choices, since the graph-theoretic null held at uncorrected as well as corrected thresholds and therefore cannot be attributed to conservative multiple-comparison correction.

Treating each graph-theoretic test as a two-sample comparison with *n*_1_ = 94 and *n*_2_ = 920, the analysis had approximately 80% power to detect a standardized between-group difference of Cohen's *d*≈0.30 (equivalently |β|≈0.09) at α = 0.05, and 90% power for *d*≈0.35. The observed cannabis-dependence effects fell below this threshold, reaching at most 0.13 standard deviations for visual-network participation in the primary analysis, and were substantially smaller than the behavioral group differences that survived correction in the same participants (*d* = 0.28–0.75).

We therefore characterize this result as an absence of a *detectable* association between cannabis dependence and resting-state network organization under the present analysis and at the present sample size. Effects that are smaller than *d*≈0.30, that are confined to subpopulations such as early-onset or very heavy users, or that reside in properties not captured by edgewise contrasts or system-level summary metrics, would not have been recovered here.

These null connectome findings are consistent with the study's central result. The structural equation models showed that variation in psychiatric symptom burden was carried substantially by psychosocial distress and only weakly by cannabis exposure, and the connectome analyses extend this pattern to the neural level where cannabis dependence was not associated with detectable reorganization of resting-state functional architecture in the unintoxicated state. The most parsimonious reading consistent with these data is that no durable, whole-brain functional-connectivity imprint of cannabis dependence was detectable at the level of diagnostic status in this sample and at this sample size. We emphasize that this interpretation is limited by the cross-sectional design, by the absence of information on recency and lifetime intensity of use, and by the possibility that effects exist in properties not captured by edgewise differences or system-level summary metrics, or in subpopulations such as early-onset or very heavy users.

The implications of these findings for clinical practice should be considered conservatively. This is a cross-sectional, observational study that contains no intervention, treatment comparison, nor longitudinal follow-up, and it therefore cannot establish that acting on any of the factors modeled here would change psychiatric outcomes. A stronger concurrent association between psychosocial distress and symptom burden does not by itself imply that treating distress would yield greater clinical benefit than addressing cannabis use, since the magnitude of an observed association and the modifiability of the underlying construct are separate questions. What the present results do support is that psychosocial context, socioeconomic disadvantage and familial risk account for a substantially larger share of the explained variance in psychiatric symptom burden than cannabis exposure does, and that clinical assessments and future research designs should therefore characterize that context alongside cannabis exposure rather than treating exposure in isolation. Whether interventions targeting stress regulation, such as cognitive-behavioral therapy for insomnia (Alimoradi et al., 2022), mindfulness-based stress reduction (Grossman et al., 2004), or structured exercise programmes (Yang and Chen, 2018), would in fact reduce symptom burden in this population is a question for prospective trials, which these data can motivate but cannot answer. Finally, because cannabis-dependent participants in this sample reported poorer outcomes on several measures, the data provide no support for a net beneficial effect of heavy cannabis use on wellbeing.

## 5 Limitations

Several limitations warrant consideration. First, all findings are cross-sectional and should be interpreted as associations rather than causal effects. Reverse causality is plausible: individuals with pre-existing externalizing tendencies may self-select into heavy cannabis use, and those with greater psychosocial distress may both use cannabis more heavily and report more symptoms. Second, the ≥1, 000 lifetime uses threshold is a proxy for chronicity that does not capture potency, route of administration, THC-to-CBD ratio, or recency of use. While the convergence of results using DSM-IV cannabis dependence as an alternative classification supports robustness, these operationalizations capture overlapping but non-identical populations. Third, the HCP sample comprises relatively healthy, well-functioning young adults recruited with an intentional twin/sibling enrichment; findings may not generalize to clinical populations, older adults, or individuals with more severe substance use disorders. Fourth, the latent Psychosocial Distress factor combines perceived stress, life satisfaction, and sleep quality into a single composite. While empirically coherent, this approach prioritizes shared variance and may obscure indicator-specific associations; for instance, sleep disruption may relate to cannabis use through distinct mechanisms such as withdrawal or altered circadian rhythms not captured by the general distress dimension. Fifth, although random intercepts for family ID partially address the HCP's twin/sibling design, shared genetic and environmental confounding cannot be fully disentangled in cross-sectional data.

Sixth, the two focal constructs are not measured with equivalent precision or on an equivalent time scale. Psychosocial distress is a continuous latent variable estimated from three current self-report indicators, whereas cannabis exposure is a binary classification based on lifetime DSM-IV dependence or a lifetime-occasions threshold. A continuous, multi-indicator latent variable is measured with less error than a dichotomy, and attenuation due to measurement error is correspondingly greater for the cannabis term; part of the difference in standardized coefficients is therefore potentially attributable to measurement properties rather than to the constructs themselves. Compounding this, the distress indicators and the ASR outcomes are both current self-report instruments and plausibly share method variance, and the constructs themselves overlap conceptually, so the distress–outcome path is potentially inflated relative to a comparison between constructs measured by independent methods. For these reasons the larger standardized coefficient for distress should not be read as demonstrating a larger biological or etiological contribution, and we have avoided that reading throughout.

Seventh, head motion was addressed by covariate adjustment rather than in preprocessing. Although the HCP minimal preprocessing pipeline incorporates ICA-FIX denoising, residual motion-related variance is known to persist after such correction and to bias functional-connectivity estimates in ways that can differ systematically between groups (Satterthwaite et al., 2013). Mean framewise displacement did not differ between the dependent and comparison groups, and adding it as a covariate left both connectome analyses unchanged, but the connectomes were not censored for high-motion frames, and adjustment for a participant-level summary cannot remove motion-related variance at the level of individual frames or edges. A replication in which motion is addressed during preprocessing, by frame censoring or by edge-level motion regression, would provide a stronger test.

Eighth, the choice of permutation scheme in the network-based statistic is an important consideration, since cannabis dependence varies largely between families (rather than within). Only 67 of the 430 families in the analytic sample contain both dependent and non-dependent members, so a within-family scheme reshuffles the exposure for just 194 of 1,014 participants. We therefore report the analysis under two schemes in the Results. The within-family null is narrower than the alternatives, which makes it less conservative. The corrected *p*-values does agree across schemes and the observed component is far from significance, so the null result does not depend on this choice.

Ninth, the outcomes carried into the structural equation and connectome analyses were selected on the basis of group comparisons conducted in the same sample, and the SEM specification itself was arrived at by iterative development on those same data. The analysis is model-generating in both respects, so the SEM estimates are not independent of the data that shaped them and should be treated as exploratory; the supplementary fit across all 23 candidate outcomes is provided so that readers can judge the sensitivity of the conclusions to that choice.

Finally, the mixture analyses fall short of demonstrating that the sample is homogeneous with respect to cannabis. For Critical Items the multi-class negative-binomial models could not be estimated, and for antisocial traits the two-class solution favored by BIC was attributed to the T-score floor, a judgement that we believe is defensible but that a reader may weigh differently, so that these analyses indicate at most that the fitted models did not identify a robust and interpretable subgroup, not that none exists. Mixture-model class recovery depends on component separation, sample size within the exposed group, and the correctness of the assumed component distribution, and a subgroup constituting a small fraction of 94 dependent participants would be difficult to recover under any of these specifications.

## Data Availability

Publicly available datasets were analyzed in this study. This data can be found here: https://humanconnectome.org.
