# Supplementary material for: Psychosocial distress is a stronger correlate of psychiatric symptom burden than cannabis dependence in young adults: a functional connectome and structural equation modeling study
Source: Front Neurosci. 2026 Oct 28;20:1922823. doi: 10.3389/fnins.2026.1922823 (PMC13751524; doi:10.3389/fnins.2026.1922823)
Supplement: Supplementary file 1 [file Data_Sheet_1.pdf]

Supplementary S1 — Complete lavaan syntax

All structural equation models reported in the manuscript use the single specification below, applied identically across the three outcomes and both exposure definitions. It was reached by iterative model development from a theory-implied mediation model (the model-generating situation of Jöreskog, 1993), and the specification that resulted frees no residual covariances: candidates among the self-report indicators were evaluated during development against modification indices and residual correlations, and none met the substantive common-method threshold set for retention. No path is trimmed after fitting.

Model specification

With `<OUTCOME>` one of `ASR_Crit_Raw`, `ASR_Totp_T`, `DSM_Antis_T`, and `<EXPOSURE>` one of `MJ_dependent` (DSM-IV dependence) or `MJ_high` ($\geq$1,000 lifetime occasions), both coded 0/1:

```
# measurement model
Stress =~ 1*PercStress_z + LifeDissat_z + SleepDebt_z

# structural model
<OUTCOME> ~ c1*<EXPOSURE> + b1*Stress + SES_z + Age_in_Yrs + Gender +
            PolySub_z + Alc_z + Nicotine_z + FamRisk_z

Stress ~ a1*<EXPOSURE> + SES_z + Age_in_Yrs + FamRisk_z

# derived effects
indirect := a1*b1
direct   := c1
total    := c1 + a1*b1
```

Estimation

```
fit <- lavaan::sem(model,
                   data      = d,
                   estimator = "MLR",          # robust ML
                   cluster   = "Family_ID")    # cluster-robust SEs by family
```

Missing data are handled by listwise deletion (lavaan's default; `missing` is not set), giving $N = 1{,}010$ of 1,016. Refitting all six models with `missing = "ml"` (full-information maximum likelihood) recovers $N = 1{,}014$ and changes every standardized total effect by at most 0.0006, with no change to any inference.

Notes on the indicators

Term	Definition	Direction
`PercStress_z`	NIH Toolbox Perceived Stress, z-scored. Loading fixed at 1 to set the latent metric.	higher = more stress
`LifeDissat_z`	NIH Toolbox Life Satisfaction, z-scored then sign-reversed.	higher = less satisfied
`SleepDebt_z`	PSQI global score, z-scored. Despite the variable name this is sleep quality, not a sleep-debt derivation.	higher = poorer sleep

Note. Covariate construction, scaling and direction are given in Table 5 of the manuscript.
