# Supplementary material for: Psychosocial distress is a stronger correlate of psychiatric symptom burden than cannabis dependence in young adults: a functional connectome and structural equation modeling study
Source: Front Neurosci. 2026 Oct 28;20:1922823. doi: 10.3389/fnins.2026.1922823 (PMC13751524; doi:10.3389/fnins.2026.1922823)
Supplement: Supplementary file 2 [file Data_Sheet_2.pdf]

Supplementary S2 — Structural equation models across all candidate outcomes

Each row refits the manuscript's single SEM specification (Supplementary S1) with a different outcome, holding the measurement model, covariate set and estimator fixed. Perceived stress and life satisfaction are indicators of the latent distress factor and are necessarily excluded as circular, leaving 21 of the 23 screened outcomes. Outcomes marked † are the three carried into the main analysis.

Outcome	Cannabis (total)		Psychosocial distress		R^2
	β	p	β	p	
Panel A. Exposure = DSM-IV cannabis dependence					
ASR Critical Items†	0.130	.001	0.595	<.001	0.438
Fluid intelligence (PMAT)	0.104	.002	-0.066	.081	0.129
Hyperactivity	0.084	.051	0.387	<.001	0.182
ADHD problems	0.082	.076	0.460	<.001	0.256
Antisocial traits†	0.066	.171	0.412	<.001	0.241
Global psychopathology (ASR Total)†	0.052	.127	0.699	<.001	0.518
Inattention problems	0.046	.280	0.452	<.001	0.224
Life has meaning and purpose	0.045	.202	-0.747	<.001	0.514
Cognitive flexibility	0.044	.230	-0.086	.027	0.067
ASR Aggressive behaviour	0.035	.488	0.531	<.001	0.310
Anxiety	0.034	.415	0.661	<.001	0.427
Somatic problems	0.030	.482	0.394	<.001	0.177
Impulsivity (DD $40k)	0.025	.516	-0.063	.106	0.058
Positive affect	0.023	.576	-0.767	<.001	0.537
Episodic memory	-0.020	.603	-0.150	<.001	0.073
Depressive problems	0.013	.740	0.693	<.001	0.458
ASR Internalizing	0.013	.714	0.762	<.001	0.558
Inhibition (Flanker)	-0.008	.815	-0.009	.836	0.051
Avoidant personality problems	0.002	.947	0.503	<.001	0.271
Impulsivity (DD $200)	0.002	.954	-0.079	.050	0.065
Self efficacy	0.001	.974	-0.597	<.001	0.364
Panel B. Exposure = heavy use (≥1,000 lifetime occasions)					
ASR Critical Items†	0.185	<.001	0.593	<.001	0.451
Antisocial traits†	0.093	.038	0.411	<.001	0.244
Hyperactivity	0.072	.086	0.386	<.001	0.181
ASR Aggressive behaviour	0.067	.159	0.530	<.001	0.312
Self efficacy	0.063	.089	-0.598	<.001	0.367
Global psychopathology (ASR Total)†	0.060	.101	0.698	<.001	0.519
Inhibition (Flanker)	-0.053	.108	-0.008	.841	0.054
ADHD problems	0.051	.265	0.460	<.001	0.253
Somatic problems	0.049	.283	0.393	<.001	0.178
Impulsivity (DD $200)	0.049	.193	-0.079	.050	0.067
Anxiety	0.048	.250	0.661	<.001	0.428
Life has meaning and purpose	0.044	.196	-0.747	<.001	0.516
Depressive problems	0.042	.321	0.693	<.001	0.459
Impulsivity (DD $40k)	-0.040	.261	-0.063	.104	0.059
Avoidant personality problems	0.038	.354	0.503	<.001	0.272
ASR Internalizing	0.037	.343	0.762	<.001	0.559
Episodic memory	-0.031	.419	-0.150	<.001	0.073
Positive affect	-0.024	.526	-0.767	<.001	0.537
Inattention problems	0.015	.714	0.451	<.001	0.223
Cognitive flexibility	-0.003	.945	-0.087	.026	0.065
Fluid intelligence (PMAT)	-0.001	.981	-0.068	.076	0.121

Note. Ordered by absolute cannabis total effect within panel. All models estimated with robust maximum likelihood and cluster-robust standard errors by family.

Summary: the cannabis total effect reached $p < .05$ for 2 of 21 outcomes under each exposure definition, whereas the distress path reached $p < .05$ for 17 of 21. The absolute distress coefficient exceeded the absolute cannabis coefficient for 20 of 21 outcomes. Median absolute cannabis effect was 0.034 (dependence) and 0.048 (heavy use), against 0.066 and 0.093 among the three outcomes selected for the main analysis — so selection inflated the cannabis estimates roughly twofold. The single outcome for which cannabis was the stronger predictor is fluid intelligence, where the distress path is itself null and where the cannabis association is positive, the dependent group scoring higher.
