# Supplementary material for: Psychosocial distress is a stronger correlate of psychiatric symptom burden than cannabis dependence in young adults: a functional connectome and structural equation modeling study
Source: Front Neurosci. 2026 Oct 28;20:1922823. doi: 10.3389/fnins.2026.1922823 (PMC13751524; doi:10.3389/fnins.2026.1922823)
Supplement: Supplementary file 3 [file Data_Sheet_3.pdf]

Supplementary S3 — Sensitivity of the network-topology null to thresholding and metric definition

To establish that the null result is not an artifact of the thresholding and efficiency definitions described in the Methods, we recomputed all network metrics for all 1,016 participants under four schemes and refitted the mixed models in each. The first scheme is the absolute-threshold computation used for the primary analysis. The remaining three apply proportional thresholding at fixed densities of 0.10, 0.20 and 0.30, retain positive weights, and convert connection strength to distance by the reciprocal transform before computing path lengths. Efficiency values under the three proportional schemes fall below unity as they should (whole-brain mean 0.24), in contrast to the absolute-threshold scheme, which averaged 1.57.

The recomputation is faithful to the primary analysis: under the absolute-threshold scheme the visual-network global efficiency test returns $p = .087$, against the $p = .086$ of Table 2. The coefficients in this section are reported on a standardized outcome scale, so that a coefficient is the between-group difference in standard deviation units, and are not directly comparable in magnitude with those in Table 2, which are expressed in raw metric units.

The null result was unchanged under every scheme. Across the 18 tests per scheme, no effect survived Benjamini-Hochberg correction in any of the four (all $p_{adj} > .09$), and only a single test of the 72 reached nominal significance before correction: limbic local efficiency at a density of 0.20 ($\beta = -0.41$, $p = .005$, $p_{adj} = .093$). We do not interpret this. It does not survive correction, it does not replicate at the adjacent densities of 0.10 or 0.30, and the limbic subnetwork is the least stable of the seven, being undefined for between 6.6% and 58.1% of participants depending on the density at which the network is thresholded. The whole-brain metrics were uniformly null across schemes, with standardized coefficients of -0.034 to -0.025 for global efficiency, -0.057 to 0.040 for local efficiency, and -0.084 to -0.011 for modularity, none approaching significance.

The conclusion that cannabis dependence is not associated with detectable differences in resting-state network topology therefore does not depend on the thresholding rule, on the density at which the graphs are constructed, or on the correction of the distance transform. It should still be read, as stated in the Limitations, as the absence of a detectable association at this sample size rather than as evidence that no association exists.
